# DRd方案治疗高龄、虚弱、高危POEMS综合征1例

**DOI:** 10.3760/cma.j.cn121090-20260225-00104

**Published:** 2026-06

**Authors:** 敏 王, 钰 仇, 振玲 李

**Affiliations:** 中日友好医院血液科，北京 100029 Department of Hematology, China-Japan Friendship Hospital, Beijing 100029, China

患者，女，78岁，因“乏力1个月余，加重伴腹胀及水肿1周”于2024年8月入院。患者入院前1个月余出现乏力，近1周乏力加重，伴右侧乳房、右侧躯干及右下肢水肿，伴腹胀及右上腹压痛。入院时ECOG评分4分，平车推入病房，全身膝关节以上褐色色素沉着，白甲，右下肺呼吸音低，腹部膨隆，移动性浊音阳性，双下肢凹陷性水肿，四肢肌力Ⅳ级，四肢痛觉呈手套样、袜套样减退。总体神经病变限制量表（ONLS）评分11分（上肢4分，下肢7分）。实验室检查：血清蛋白电泳：M蛋白阴性；血清免疫固定电泳：IgA-λ型M蛋白；尿免疫固定电泳：阴性；血清血管内皮生长因子（VEGF）：992.38（正常值：0～142）pg/ml。胸、腹、盆腔CT：肺内渗出灶，心包及两侧胸腔积液，腹盆腔积液，皮下水肿，胆囊结石。心脏超声：左房扩大，室间隔稍厚（12 mm），左室射血分数71％，肺动脉压力55 mmHg；肌电图：上下肢周围神经神经源性损害。骨髓形态、免疫分型及病理均未见异常浆细胞。根据POEMS综合征的诊断标准，患者满足2条强制标准（多发周围神经病变、M蛋白血症），1条主要标准（VEGF水平升高），3条次要标准（血管外体液容量增加、内分泌异常、皮肤改变），POEMS综合征诊断明确。该患者78岁高龄，ECOG评分4分，卧床状态，合并症多，老年评估为虚弱。2024年8月28日开始予减量Rd方案治疗（来那度胺10 mg，第1～14天；地塞米松20 mg，第1、8、15、22天）。治疗后，患者肌酐进行性升高，最高升至232.7 µmol/L，患者乏力、肢体麻木及水肿症状无改善。2024年9月在原方案基础上加用达雷妥尤单抗（DRd方案，达雷妥尤单抗16 mg/kg，静滴，前8周每周输注1次；第9～24周每2周输注1次；25周以后每4周输注1次），根据估算肾小球滤过率（eGFR）调整来那度胺剂量。DRd方案治疗后，患者肌酐水平逐渐恢复正常，乏力及手脚麻木症状好转，皮肤色素沉着及白甲减轻，食欲改善，全身水肿减轻，多浆膜腔积液消失。2025年2月患者能够自行站立及扶助步器行走，ONLS评分6分（上肢2分，下肢4分）。2024年11月、2025年4月患者免疫固定电泳及蛋白电泳均阴性，达到血液学完全缓解（CR_H_）；2024年11月、2025年4月患者血清VEGF分别为180 pg/ml、109 pg/ml，达到VEGF完全缓解（CR_V_）。患者第1次输注达雷妥尤单抗时出现胸闷、气短，血氧饱和度未降低，考虑Ⅰ级输注反应，暂停输液。予盐酸异丙嗪及地塞米松处理后，患者症状好转，未再出现输注反应；患者出现粒细胞缺乏伴发热1次，接受升白细胞、抗感染治疗后好转。2025年2月患者出现右上腹痛，诊断为急性胆囊炎，行胆囊穿刺置管引流术，2025年4月行腹腔镜下胆囊切除，术后恢复可。患者高龄，合并急性胆囊炎，腹腔镜下胆囊切除术后，POEMS综合征经治疗后临床症状明显改善，达到CR_H_及CR_V_，故停用达雷妥尤单抗输注，患者共接受DRd方案治疗6个周期。胆囊炎期间及胆囊炎治疗后口服来那度胺维持治疗6个周期后停止治疗。患者每3～6个月复查随访，病情均处于稳定状态。

讨论：北京协和医院李剑团队的研究显示，年龄>50岁、eGFR<30 ml·min^−1^·（1.73 m^2^）^−1^、肺动脉高压、胸腔积液为POEMS综合征的不良预后因素，有2个及以上危险因素为高危。本例患者有3个危险因素，危险分层为高危。患者最初应用减量Rd方案治疗后出现肌酐进行性升高，考虑与Rd方案起效慢、患者病情重相关，加用达雷妥尤单抗后，患者肌酐恢复正常，1个疗程后临床症状明显改善，3个疗程后达到CR_H_，6个疗程后达到临床缓解，9个月评估时达到CR_V_，初步提示对于高危、高龄、病情进展快、虚弱的患者，可考虑一线应用含达雷妥尤单抗的方案治疗。

